# A Milestone in the Chemical Synthesis of Fe_3_O_4_ Nanoparticles: Unreported Bulklike Properties Lead to a Remarkable Magnetic Hyperthermia

**DOI:** 10.1021/acs.chemmater.1c02654

**Published:** 2021-11-10

**Authors:** Idoia Castellanos-Rubio, Oihane Arriortua, Daniela Iglesias-Rojas, Ander Barón, Irati Rodrigo, Lourdes Marcano, José S. Garitaonandia, Iñaki Orue, M. Luisa Fdez-Gubieda, Maite Insausti

**Affiliations:** † Dpto. Electricidad y Electrónica, Facultad de Ciencia y Tecnología, UPV/EHU, Barrio Sarriena s/n, 48940 Leioa, Spain; ‡ Dpto. Química Inorgánica, Facultad de Ciencia y Tecnología, UPV/EHU, Barrio Sarriena s/n, 48940 Leioa, Spain; § BC Materials, Basque Center for Materials, Applications and Nanostructures, Barrio Sarriena s/n, 48940 Leioa, Spain; ∥ Helmholtz-Zentrum Berlin für Materialien und Energie, Albert-Einstein-Str.15, 12489 Berlin, Germany; ⊥ Dpto. Física Aplicada II, Facultad de Ciencia y Tecnología, UPV/EHU, Barrio Sarriena s/n, 48940 Leioa, Spain; # SGIker, Servicios Generales de Investigación, UPV/EHU, Barrio Sarriena s/n, 48940 Leioa, Spain

## Abstract

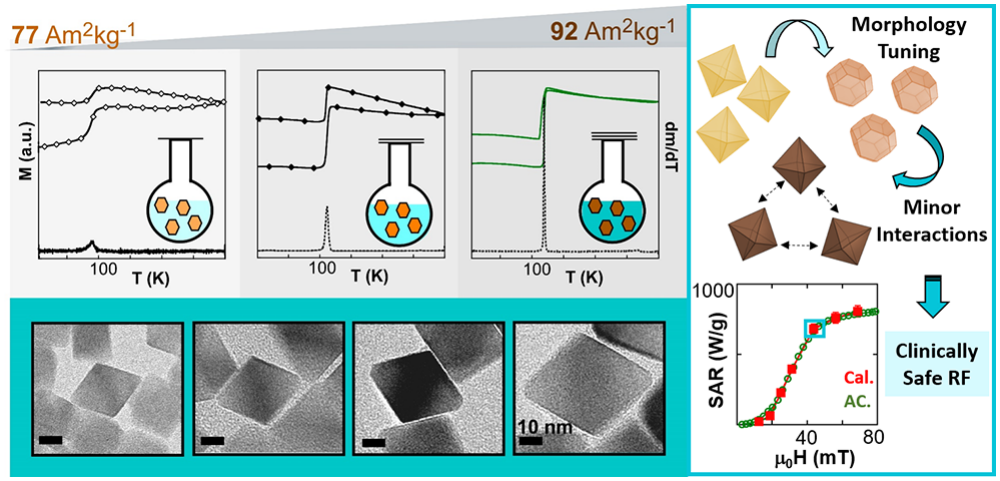

Among iron oxide phases, magnetite (Fe_3_O_4_) is often the preferred one for nanotechnological and biomedical applications because of its high saturation magnetization and low toxicity. Although there are several synthetic routes that attempt to reach magnetite nanoparticles (NPs), they are usually referred as “IONPs” (iron oxide NPs) due to the great difficulty in obtaining the monophasic and stoichiometric Fe_3_O_4_ phase. Added to this problem is the common increase of size/shape polydispersity when larger NPs (*D* > 20 nm) are synthesized. An unequivocal correlation between a nanomaterial and its properties can only be achieved by the production of highly homogeneous systems, which, in turn, is only possible by the continuous improvement of synthesis methods. There is no doubt that solving the compositional heterogeneity of IONPs while keeping them monodisperse remains a challenge for synthetic chemistry. Herein, we present a methodical optimization of the iron oleate decomposition method to obtain Fe_3_O_4_ single nanocrystals without any trace of secondary phases and with no need of postsynthetic treatment. The average dimension of the NPs, ranging from 20 to 40 nm, has been tailored by adjusting the total volume and the boiling point of the reaction mixture. Mössbauer spectroscopy and DC magnetometry have revealed that the NPs present a perfectly stoichiometric Fe_3_O_4_ phase. The high saturation magnetization (93 (2) A·m^2^/kg at RT) and the extremely sharp Verwey transition (at around 120 K) shown by these NPs have no precedent. Moreover, the synthesis method has been refined to obtain NPs with octahedral morphology and suitable magnetic anisotropy, which significantly improves the magnetic hyperthemia performance. The heating power of properly PEGylated nano-octahedrons has been investigated by AC magnetometry, confirming that the NPs present negligible dipolar interactions, which leads to an outstanding magnetothermal efficiency that does not change when the NPs are dispersed in environments with high viscosity and ionic strength. Additionally, the heat production of the NPs within physiological media has been directly measured by calorimetry under clinically safe conditions, reasserting the excellent adequacy of the system for hyperthermia therapies. To the best of our knowledge, this is the first time that such bulklike magnetite NPs (with minimal size/shape polydispersity, minor agglomeration, and exceptional heating power) are chemically synthesized.

## 1 Introduction

In the last two decades, iron oxide nanoparticles (IONPs) have become increasingly versatile building blocks for a wide range of applications that encompass very diverse fields such as biomedicine,^1,2^ magnetic data storage,^3^ and environmental remediation.^4^ Specifically, the number of works that make use of IONPs for therapy and diagnosis, including magnetic hyperthermia, magnetic resonance imaging, and drug delivery among others, increase in number every year.^5^ From a chemical point of view, the standardized use of “IONPs” might seem quite vague given that there are multiple iron oxide polymorphs that exhibit an entirely distinct set of properties.^6^ Certainly, application-specific performance metrics completely depend on the nanoparticle’s crystal structure and composition. The most common form of iron oxide in nature is hematite (α-Fe_2_O_3_) with a corundum structure and canted antiferromagnetism at room temperature (RT).^7^ However, the most interesting and demanding iron oxide phases for nanotechnological applications are mixed-valence magnetite (Fe_3_O_4_) and fully oxidized maghemite (γ-Fe_2_O_3_), both with an inverse spinel and ferrimagnetic order.^8^ Indeed, magnetite and maghemite nanoparticles are among the most widely used nanosystems due to their good magnetic performance and low toxicity,^9,10^ magnetite being the preferred option because of its higher saturation magnetization (*M*_s_ = 92 A·m^2^/kg at RT).^11−13^ Unfortunately, even optimal synthesis routes that offer the best control over the particle size/shape while minimizing particle polydispersity usually produce a mixture of nonstoichiometric maghemite and magnetite phases.^14−16^ Moreover, decomposition of iron oleate, which is considered a matchless wet-chemical method to finely adjust IONP size over 20 nm, commonly leads to biphasic nanoparticles composed of magnetite and metastable wüstite (Fe_*x*_O, *x* = 0.83–0.96).^17−19^ Wüstite is a complex nonstoichiometric oxide that compositionally lies between pure FeO and Fe_3_O_4_; it crystallizes in a NaCl-type defective structure and shows weak paramagnetism at RT.^20^ Obviously, the presence of this phase in the IONP system causes a serious degradation of the magnetic properties.^21^ The great difficulty in obtaining monophasic IONPs explains why the imprecise “IONP” nomenclature has been universally accepted. In the last few years, some new strategies have been developed to remove the wüstite phase from the NPs,^22,23^ but even the IONPs produced by the most sophisticated chemical methods are still quite far from reaching stoichiometric Fe_3_O_4_ NPs showing bulklike properties.^24,25^ To date, there has been no record of synthetically produced magnetite nanoparticles that present *M*_s_ equal to the bulk material, i.e., 92 A·m^2^/kg at RT and 98 A·m^2^/kg at 5 K. So far, the magnetite nanocrystals with the highest purity have been produced by magnetotactic bacteria through a surprisingly perfect biomineralization process orchestrated by a genetic blueprint information encoded in their genome.^26^ So the question is: would it be possible to chemically synthesize magnetite nanoparticles as good as or even better than the ones that magnetotactic bacteria have kept refining since the Cambrian Period?

There is no doubt that application-centered research is appealing and profitable, and it is undeniable, likewise, that the success of any technological and biomedical application relies on the quality of the material used. Thus, we have taken up the challenge and have brought the optimization of magnetite nanoparticles one step further. Herein, we present a one-pot synthetic strategy based on the pyrolysis of iron oleate to prepare magnetite nanoparticles with unprecedented high saturation magnetization. These NPs (with sizes ranging from 20 to 40 nm) are single crystals and present perfect Fe_3_O_4_ stoichiometry, which has been reliably determined by Mössbauer spectroscopy. Additionally, the synthesis method has been refined in order to obtain nanoparticles with an octahedral shape, which has been proven to present a more suitable magnetic anisotropy for magnetothermal actuation than other morphologies.^27,28^ The magnetothermal efficiency of properly PEGylated nano-octahedrons has been investigated in aqueous colloids, in physiological solution, and in agar, reaching exceptional and identical specific absorption rate (SAR) values in the three media and under sanitary RF field restrictions. To the best of our knowledge, this is the first time that such excellent quality magnetite NPs are chemically synthesized in terms of both crystallinity and size/shape homogeneity. The structural, chemical, and magnetic characterization of the optimal batch of samples have been carefully compared to highly considered previous batches that were recently published.^27,29^ This comparison is aimed at facilitating a clear display about the improvement achieved in the present Fe_3_O_4_ NPs.

## 2 Results and Discussion

### 2.1 Optimization of the Chemical Synthesis Leads to Unprecedented Magnetite Nanocrystals

**Figure 1:**
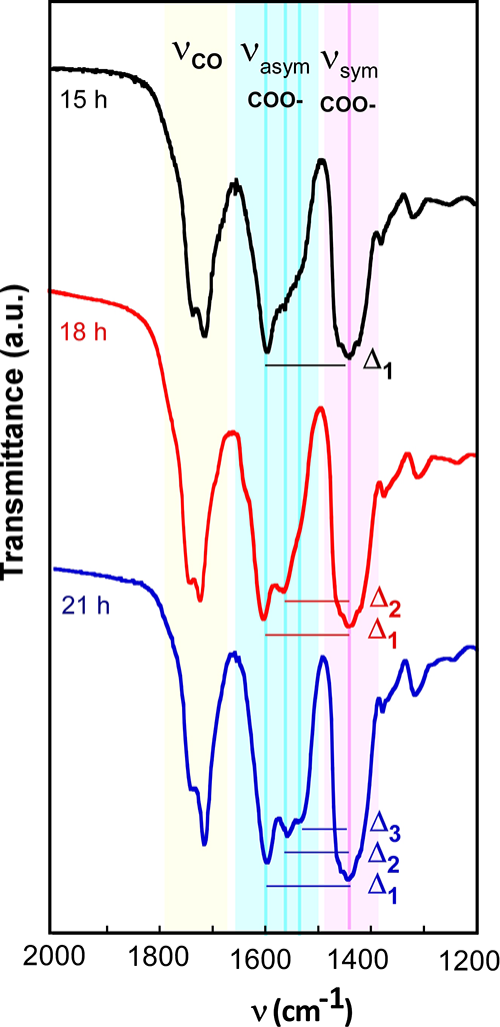
FTIR spectra of FeOl complexes dried during 15, 18, and 21 h. Evolution of the (C=O) band, asymmetric and symmetric ν(COO^–^) bands, and Δ (ν_asym_ – ν_sym_) with the drying time: Δ_1_ ≈ 150 cm^–1^ (bridging), Δ_2_ ≈ 110 cm^–1^ (bridging/bidentate), and Δ_3_ ≈ 80 cm^–1^ (bidentate). The FTIR spectra in the 4000–400 cm^–1^ range are shown in Figure S1, Supporting Information.

Although iron oleate (FeOl) is a widely used metalorganic precursor for the synthesis of IONPs, it is also a complex starting material because it can take diverse structures depending on the preparation.^30^ The FeOl complex can exist in four different metal–carboxylate coordination types (ionic, unidentate, bridging, and bidentate).^31^ The presence of different counterions and coordination solvent molecules in the FeOl can drive the nucleation and growth of the NPs to very distinct paths.^32,33,2^ In order to minimize the unpredictable effect that ionic species and remaining solvent molecules can cause, our FeOl product has been washed three times with D.I. H_2_O and dried at 110 °C overnight (see the Experimental Section). Additionally, several preparations with different drying periods have been carried out (15, 18, and 21 h) and their structural differences have been studied using Fourier Transform Infrared Spectroscopy (FTIR). Metal carboxylates display characteristic IR bands between 1500 and 1600 cm^–1^ for asymmetric stretching vibrations (blue region in Figure 1) and between 1400 and 1450 cm^–1^ for symmetric stretching vibrations (pink region in Figure 1).^34^ The distance between the asymmetric and symmetric ν(COO^–^) bands (Δ = ν_asym_ – ν_sym_) allow us to deduce the nature of the metal–carboxylate coordination. While a Δ < 110 cm^–1^ refers to a bidentate coordination mode, a Δ > 200 cm^–1^ indicates a unidentate coordination, and for intermediate values (110 cm^–1^ < Δ < 200 cm^–1^) a bridging mode is expected.^35^ On the other hand, bands at 1711 and 1736 cm^–1^ are assigned to stretching modes of the carbonyl group (C=O) (yellow region in Figure 1) of oleic acid molecules that are not coordinated or weakly coordinated in an ionic-type configuration.^36,37^ As the drying time of the FeOl is increased from 15 to 21 h, the ν_asym_(COO^–^) band slightly widens (due to the band splitting) at the expense of narrowing the (C=O) band. Consequently, the Δ separation turns out to have lower values (see Δ_2_ ≈ 110 cm^–1^ and Δ_3_ ≈ 80 cm^–1^ in Figure 1), which can be construed as a partial coordination conversion from ionic-type toward bridging and bidentate. The effect of this coordination conversion is also visible in the thermogravimetric (TG) curves of the three FeOl complexes (FeOl-15 h, FeOl-18 h, and FeOl-21 h) shown in Figure S2 of the Supporting Information. The thermal decomposition of the FeOls is moderately shifted toward higher temperatures with the increasing drying time, reaching a distinctive profile for the FeOl-21 h sample. Both FTIR and TG results indicate that weakly coordinated ligands pass to form stronger coordination as the drying time increases, which is in good agreement with prior studies.^38^

The best nanoparticle systems reported so far, in terms of homogeneity, have been obtained when the thermal window between nucleation (initiated by the dissociation of weakly bonded ligands from metal centers) and growth (dictated by the loosening of strongly coordinated ligands) was ≥80 K.^39^ In our case, the increase of the FeOl drying time to 21 h results in a suitable mixture of ionic, bridging, and bidentate coordination modes that provide a perfect decomposition window for the production of highly uniform NPs. The use of an optimized FeOl precursor together with some other synthetic refinements, detailed in the following , has led to magnetite NPs of an unparalleled quality. Table 1 shows three groups of representative samples that illustrate the continuous improvement of the magnetite phase and the synthetic parameters involved in such optimization. Samples have been named according to the optimization stage and the NP size as follows: Opt_*x*_–D_TEM,_ where *x* represents the stage (from 1 to 3) and D_TEM_ the average dimension (tip-to-tip average distance) obtained by transmission electron microscopy (TEM) analysis. Samples from groups Opt_1_ and Opt_2_ were published recently,^27,29^ while the samples from the Opt_3_ group are the focal point of the present work.

**Table 1:**
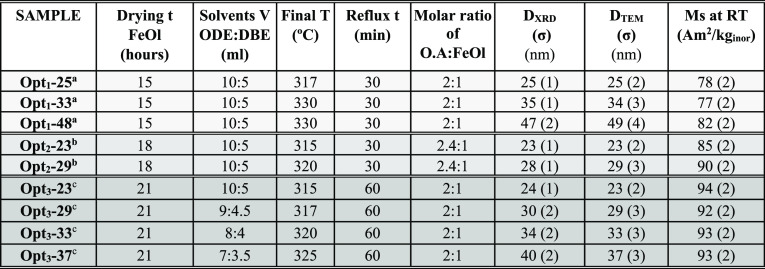
Summary of Synthesis Conditions and Samples Features Drying time of the FeOl Precursor Prior to NP Synthesis, Volumes of 1-Octadecene and Dibenzyl Ether (ODE:DBE) Used in the NP Synthesis, The Molar Ratio of Oleic Acid and FeOl (O.A:FeOl), Final Temperature, Reflux Time, Crystallite Size (*D*_XRD_) Calculated by the Scherrer Equation, Particle Mean Dimension Obtained by TEM (*D*_TEM_), and Saturation Magnetization (*M*_s_) at RT. The Three Optimization Stages are: Opt_1_, Opt_2_, and Opt_3_ (Arranged in Increasing Gray Levels)

a Samples from ref (27). Opt_1_-48 was mechanically stirred at 60 rpm and the rest of the samples at 120 rpm.

b Samples from ref (29).

c Opt_3_ batch. The reaction vessel was completely sealed up using adjustable homemade ground glass joint clips.

“Nonaqueous redox phase-tuning” is a very suitable method to avoid the formation of the wüstite phase during the nanoparticle growth process.^22^ Redox active species, coming from 1-octadecene (ODE) and dibenzyl ether (DBE) solvents, are generated during the high temperature synthesis stage. Specifically, DBE decomposition generates benzaldehyde, which possesses oxidative character;^25^ meanwhile, the tendency of the ODE’s vinyl group to oxidation produces a reductive effect.^40^ A solvent volume ratio (ODE:DBE) equal to 2:1 has proved to promote the formation of a mixed-valence magnetite phase.^27^ It is of utmost importance that the reaction system is perfectly sealed up to maintain, during the whole synthesis process, the suitable oxidative/reductive conditions that provide the formation of the magnetite phase. However, little leaks (especially from the middle neck of the flask in which a mechanical stirrer is inserted, see Figure S3 in the Supporting Information) are common due to the violent thermal decomposition of the FeOl and the strong boiling of the mixture. As small amounts of the solvent with the lowest boiling point (DBE) scape from the reaction flask, the oxidative/reductive balance is overturned toward more reductive conditions (ODE:DBE > 2:1), affecting the stoichiometry of the nanoparticles. This fact also causes a slow increase of the boiling point of the mixture, which can provoke a gradual loss of reflux and concomitant energy supply for the reaction if the final *T* (output *T*) is kept at the initial boiling point (samples Opt_1_-25 and Opt_2_-23 in Table 1). Otherwise, if the output *T* is set at higher values than what corresponds to the initial boiling point of the mixture, the leakage usually escalates worsening the oxidative/reductive balance (samples Opt_1_-33 and Opt_1_-48). In order to avoid this problem, the synthesis setup has been improved by using homemade ground glass joint clips that are precisely tightened and locked in every preparation (samples Opt_3_ in Table 1). In this way the final temperature and the reflux of the mixture have been adjusted by changing the solvent volumes while keeping ODE:DBE = 2:1. For instance, a reaction mixture with 10 mL of ODE and 5 mL of DBE has given rise to an uninterrupted reflux at 315 °C producing NPs of 23 nm (sample Opt_3_-23), and a mixture with 7 mL of ODE and 3.5 mL of DBE has resulted in a continuous 325 °C refluxing, which leads to the largest NPs of the Opt_3_ batch (sample Opt_3_–37). Additionally, the reflux time in Opt_3_ samples has been extended to 60 min to prompt a better stoichiometry in the nanoparticles.

**Figure 2:**
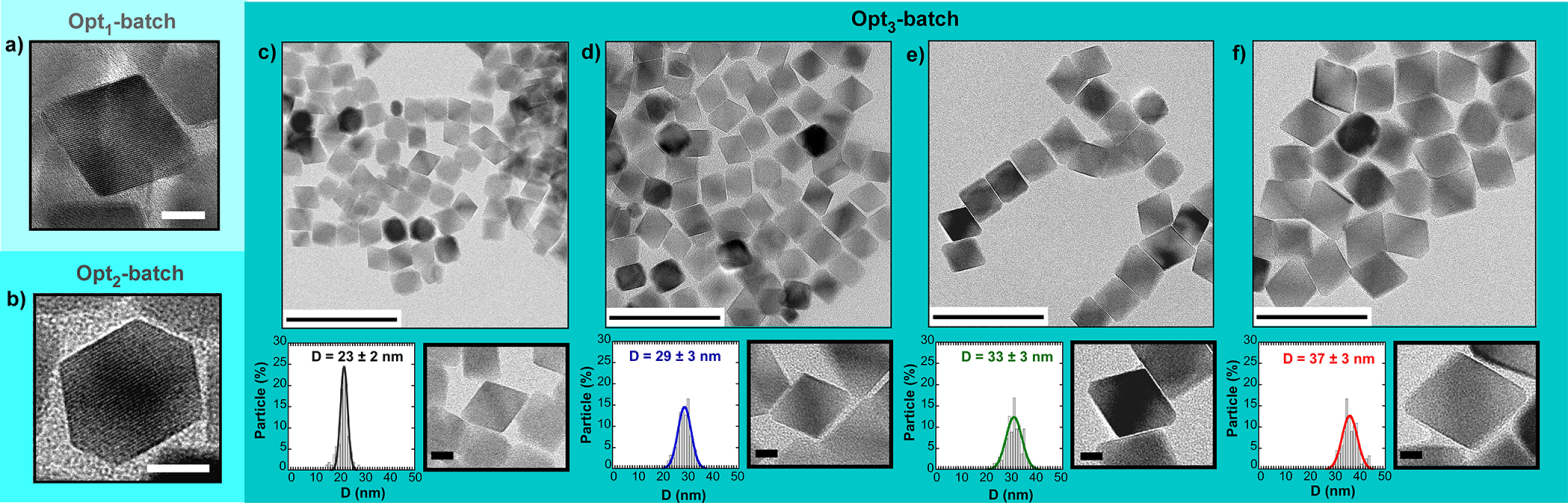
Representative nanoparticle from (a) Opt_1_ batch and (b) Opt_2_ batch (white bars: 10 nm). TEM micrographs and corresponding size distributions of the as-synthesized samples from the Opt_3_ batch: (c) Opt_3_-23, (d) Opt_3_-29, (e) Opt_3_-33, and (f) Opt_3_-37. Large scale bars: 100 nm. Zoomed-in scale bars: 10 nm. The TEM analysis of Opt_1_ and Opt_2_ batches can be found in refs (27, 29), respectively.

The detailed structural, chemical, and magnetic characterization of the samples from Opt_1_ and Opt_2_ batches can be found in our previous studies,^27,29^ respectively. The TEM micrographs of the as-synthesized Opt_3_ batch samples are displayed in Figure 2 and show monodisperse nanoparticles with sizes ranging from 23 to 37 nm. Control over the size has been attained by adjusting the final temperature; reasonably, higher final *T* translates into larger NPs (Table 1).

Additionally, the morphology has been tuned by modifying the oleic acid:FeOl molar ratio. In the cases where a lower amount of oleic acid is available during the synthesis (2:1 ratio, see Table 1), the {100} planes (which have a lower planar packing fraction in fcc structures and thus are more reactive) tend to grow to extinction promoting the formation of octahedral NPs (samples from Opt_1_ and Opt_3_ batches, see Figure 2). Conversely, the excess of oleic acid (oleic acid:FeOl ≥ 2.4:1)^27,29^ produces a steric barrier that makes the growth rate of different facets nearly equal, causing the formation of cuboctahedron-like particles (see Figure 2b).

**Figure 3:**
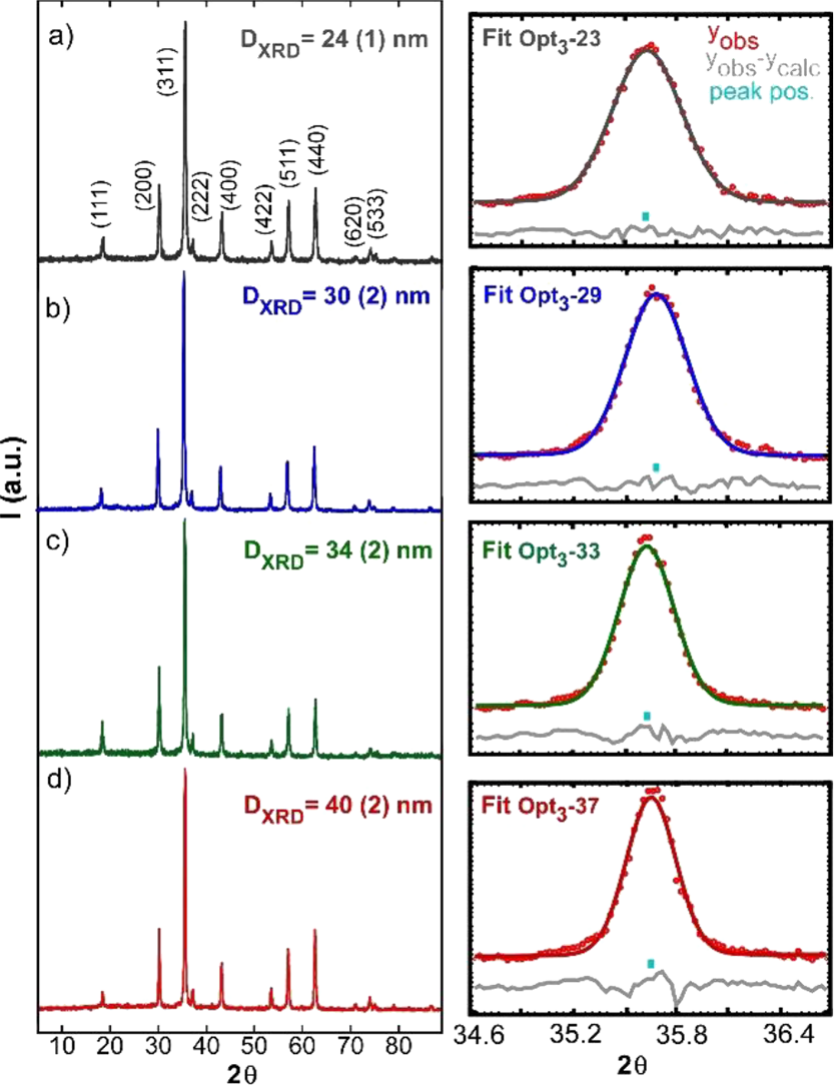
X-ray powder diffraction patterns together with the deconvolution of the main diffraction peak (311), right column, and the obtained crystallite sizes of samples (a) Opt_3_-23, (b) Opt_3_-29, (c) Opt_3_-33, and (d) Opt_3_-37. In the right column: experimental points (in red) = *y*_obs_, peak position (turquoise mark) = *peak pos*, and difference between experimental data and fit (gray line) = *y*_obs_ – *y*_calc_.

Powder X-ray diffraction (XRD) of the whole set of samples (Opt_1_, Opt_2_, and Opt_3_ batches) has not revealed any significant differences among the crystalline structure of the samples. All the samples have showed the inverse spinel structure of magnetite (PDF #880866) with no trace of wüstite or other phase. The powder diffractograms of Opt_3_ samples are displayed in Figure 3 together with the deconvolution of the (311) diffraction peak. The calculated crystalline sizes of Opt_3_ samples (see Table 1 and Table S1 in the Supporting Information) match very well with the average dimensions determined by TEM analysis, meaning that all the samples are composed of single crystals.

The immediate indicator that reflects the gradual improvement of the magnetite phase from batch Opt_1_ to Opt_3_ is the *M*_s_ of the samples at RT. As it can be observed in Table 1, the *M*_s_ increases progressively with the refinement of several synthetic parameters commented above such as the increase of FeOl drying time, adjustment of the reflux temperature, and enlargement of the final stage of the synthesis. It is remarkable that the RT *M*_s_ of samples from the Opt_3_ batch equals the bulk magnetite value (92 A·m^2^/kg), fact that advances the excellent quality of this batch of NPs. In order to gain a thorough insight into the crystalline structure and the stoichiometry of the NPs, samples from the three batches have been investigated by means of Mössbauer spectroscopy and DC magnetometry.

#### 2.1.1 Mössbauer Spectroscopy

**Figure 4:**
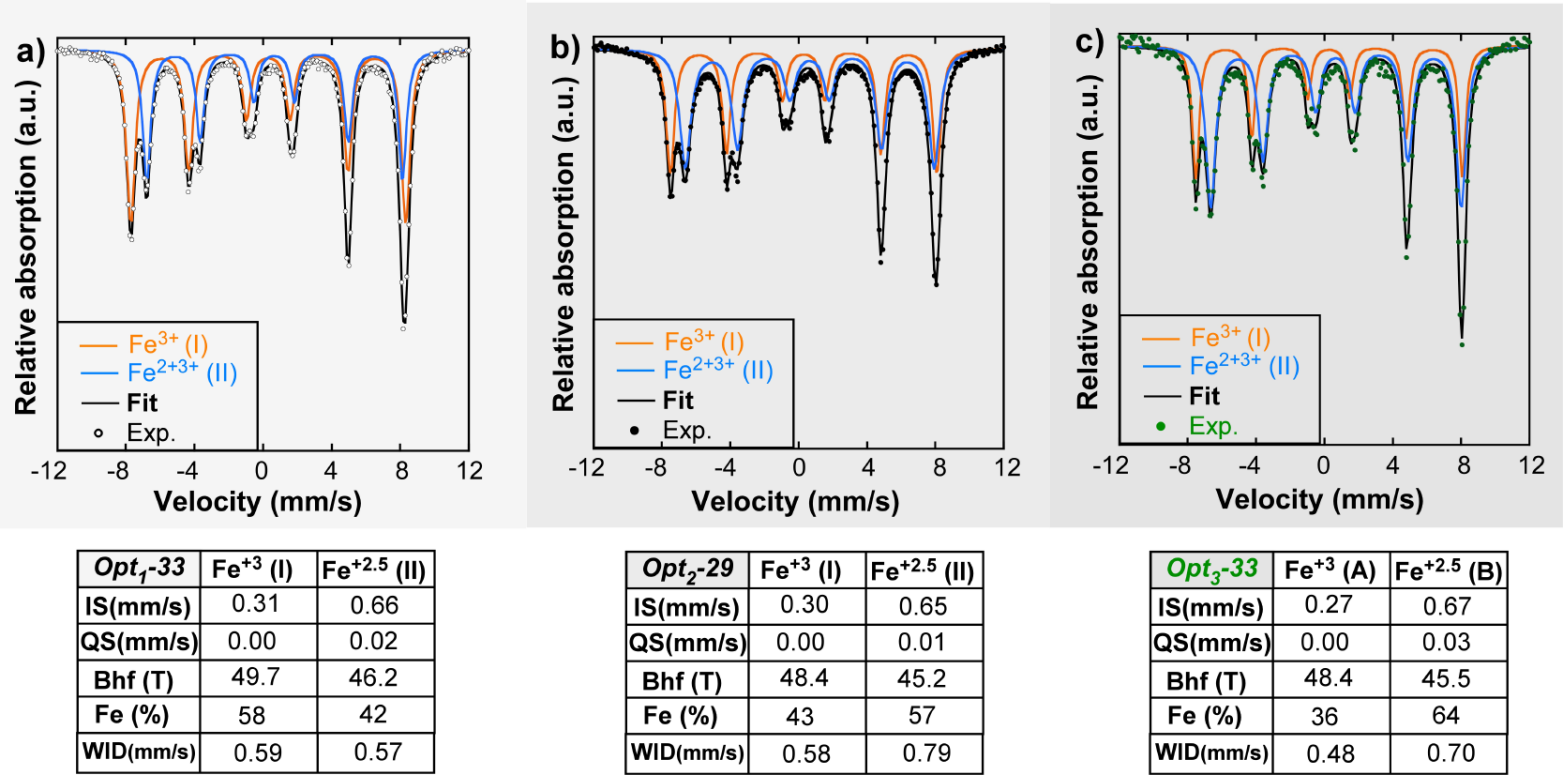
Mössbauer spectra of (a) Opt_1_-33, (b) Opt_2_-29, and (c) Opt_3_-33 NPs collected at RT together with hyperfine parameters obtained from the fittings of the spectra. IS is relative to bcc-Fe. The optimization stages of Opt_1_, Opt_2_, and Opt_3_ are arranged in increasing gray level.

If the thermal fluctuation effect is sufficiently small, a detailed analysis of the ^57^Fe Mössbauer spectra at RT allows us to study the crystallographic evolution from the Opt_1_ batch to Opt_3_ batch by analyzing the relative occupancy of Fe ions in A and B sites of the spinel lattice. Figure 4 presents the normalized Mössbauer spectra of a representative sample from each group (Opt_1_-33, Opt_2_-29, and Opt_3_-33) with a similar average dimension of 30 nm, together with the hyperfine parameters obtained from the fittings.

Briefly described, Mössbauer spectra of stoichiometric bulk Fe_3_O_4_ magnetite are composed by two well resolved sextets, being the sextet with the higher hyperfine field (I) (∼49 T) generated by Fe^3+^ ions in tetrahedral sites (A) and the one with lower hyperfine field (II) (∼46 T) assigned to Fe^2+^Fe^3+^ atoms in the octahedral (B) ones.^41^ The electron hoping among Fe^2+^ and Fe^3+^ atoms in the octahedral (B) is much faster than the resolution time of the Mössbauer spectroscopy and the contribution of this site to the spectrum is resolved by an only averaged spectral component. Therefore, it is usual to visualize this sextet as associated to only the ionic state with a Fe^2.5+^ intermediate valence representing a Fe^2+^Fe^3+^ pair of hoped atoms. The relative resonant area ratios among two components is, thus, *S*_I_/*S*_II_ = 0.5 replicating the population of both crystallographic A and B sites.

Mössbauer spectra of Opt_1_-33, Opt_2_-29, and Opt_3_-33 samples reproduce the above description, presenting similar hyperfine parameters but, as it can be seen in Figure 4, with different relative resonant areas to those expected from a stoichiometric magnetite. The calculated *S*_I_/*S*_II_ ratios of samples Opt_1_-33, Opt_2_-29, and Opt_3_-33 are 1.38, 0.75, and 0.56, respectively, evidencing the clear shift of the samples from a system with a substantial deficit of Fe ions in B sites to a stoichiometric magnetite (see Table 2).

**Table 2:**
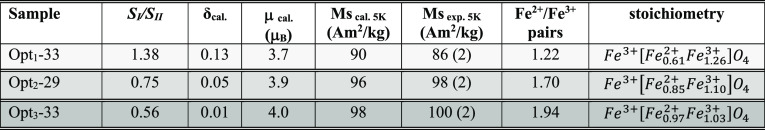
Summary of Experimental*S*_I_/*S*_II_, Calculated Fe^2+^/Fe^3+^ Pairs, Fe Vacancies (δ_cal._), Magnetic Moment (μ_cal_), and Saturation Magnetization (Ms_cal.5K_) together with the Experimental Saturation Magnetization (Ms_exp.5K_) of Samples Opt_1_-33, Opt_2_-29, and Opt_3_-33. δ_cal.,_ μ_cal._, and Fe^2+^/Fe^3+^ Pairs Have Been Calculated Using eqs 3^4^,5, Respectively. The Optimization Stages of Opt_1_, Opt_2_, and Opt_3_ Are Arranged in Increasing Gray Levels

Since the thermal decomposition of FeOl usually leads to IONPs with Fe^2+^ deficiency in B sites,^19^ the most logical approach is to assume that nonstoichiometry of the samples is related to Fe^2+^ vacancies at the octahedral position which, in order to maintain the electronic neutrality, simultaneity infers a relative increase of Fe^3+^ in this site. Formally, a nonstoichiometric magnetite with Fe^2+^ deficiency in B positions is represented by the following expression:
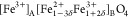
1where δ symbolizes the vacancies (0 ≤ δ ≤ 0.33).

By means of a suitable modification of eq 1, the Fe^2+^/Fe^3+^ hoped pairs and the excess of Fe^3+^ in the B crystallographic site can be easily stated as:
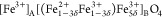
2

Therefore, following eq 2, there are 5δ unbalanced Fe^3+^ ions in the B octahedral position that do not participate in the Fe^2+^–Fe^3+^ electronic hopping and its contribution to the Mössbauer spectrum is commonly evaluated in the higher hyperfine sextet I.^42^ The *S*_I_/*S*_II_ relative resonant area ratio is, consequently, defined by eq 3:
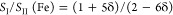
3which allows us to estimate the δ vacancy parameter. Pointing to eq 2 up again, the net magnetic moment can be calculated to be (eq 4):
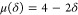
4

and the number of Fe^2+^/Fe^3+^ pairs per formula unit is determined by eq 5:
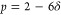
5

The number of vacancies has been considerably reduced from the Opt_1_ batch to Opt_3_ batch, which obviously is reflected in the magnetic moment (μ) and *M*_s_ of the samples. The calculated *M*_s_ at 5 K (obtained from μ_cal._) is in agreement with the experimental *M*_s_ at 5 K (see Tables 2 and 3). The corollary to this is that the progressive refinement of the synthetic protocol favors the growth of a stoichiometric magnetite phase without Fe^2+^ vacancies (δ ≈ 0), which leads to Fe_3_O_4_ NPs with truly bulklike saturation magnetization.

**Table 3:**
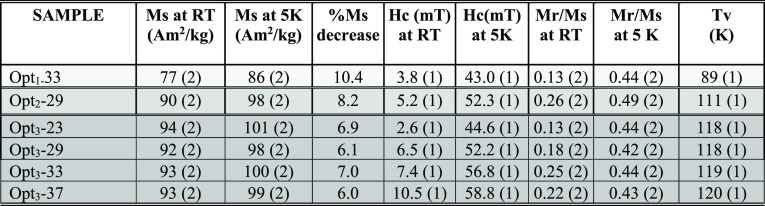
Summary of *M*_s_, Coercivity (*H*_c_), and Reduced Remanence (*M*_r_/*M*_s_) of Opt_3_ Batch Samples Obtained from the Hysteresis Loops at 300 and 5 K and the Verwey Transition Temperature (*T*_v_); the Optimization Stages of Opt_1_, Opt_2_, and Opt_3_ are Arranged in Increasing Gray Levels^a^

a Bulk magnetite 92 A·m^2^/kg at RT and 98 A·m^2^/kg at 5 K, 6.1% *M*_s_ decrease.^46^

### 2.2 Magnetic Characterization

#### 2.2.1 DC Magnetometry

**Figure 5:**
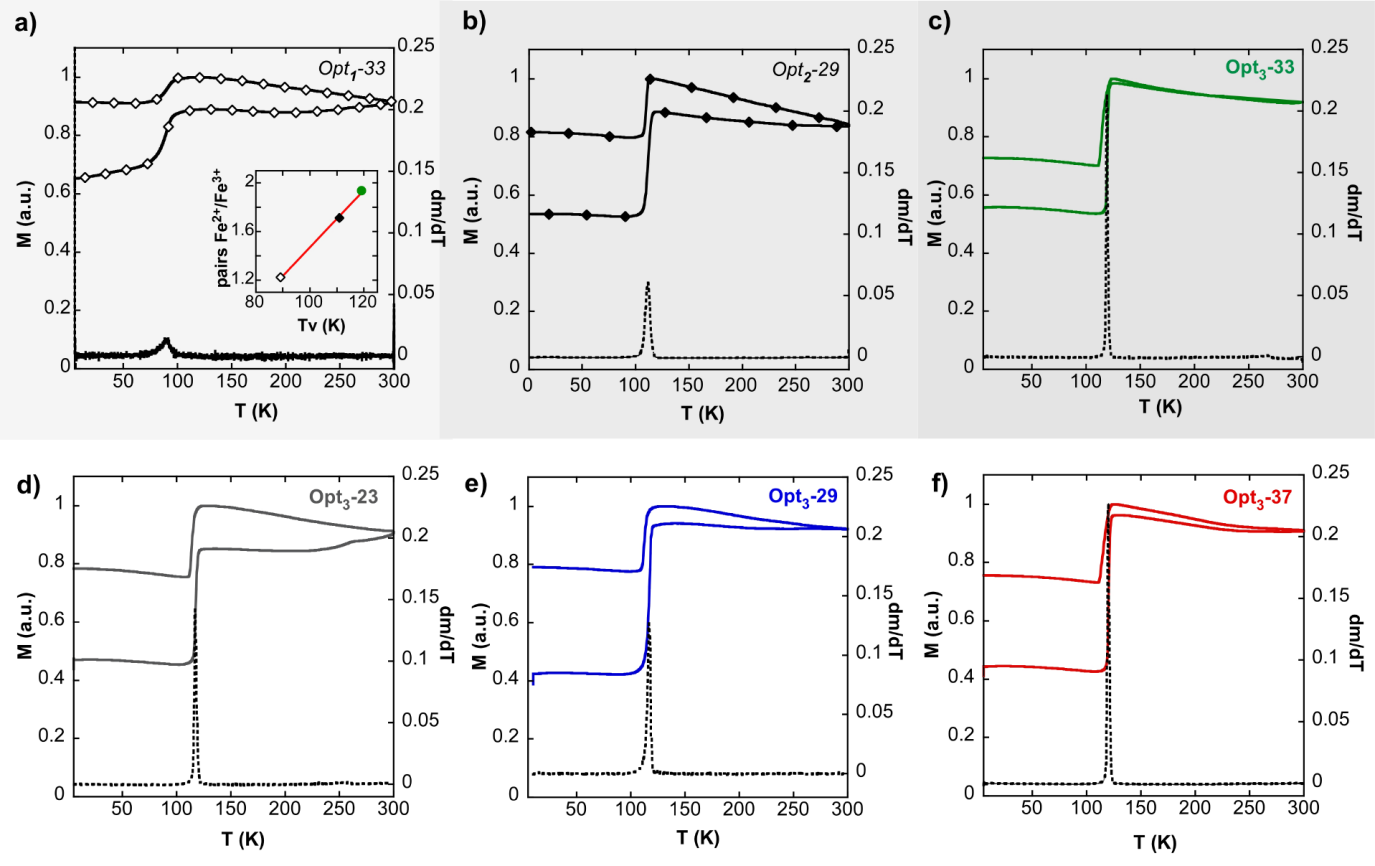
Zero field cooling and field cooling (ZFC-FC) curves together with derivatives of ZFC magnetization (black line) of samples: (a) Opt_1_-33, (b) Opt_2_-29, (c) Opt_3_-33, (d) Opt_3_-23, (e) Opt_3_-29, and (f) Opt_3_-37. Inset: Fe^2+^/Fe^3+^ pairs obtained by the analysis of Mössbauer spectra vs Verwey transition temperature (*T*_v_) of samples Opt_1_-33, Opt_2_-29, and Opt_3_-33. The optimization stages Opt_1_, Opt_2_, and Opt_3_ are arranged in increasing gray levels.

Quasistatic magnetic measurements performed under a constant temperature and field have allowed rounding off the preceding discussion and completing the portray of Opt_3_ batch samples. It is well known that magnetite crystals undergo a phase transition at around 120 K (Verway transition, *T*_v_), which produces sharp changes in the crystal lattice, the electron transport, and the magnetic properties of the system. What is relevant in this transition is that the high temperature cubic lattice changes to a low temperature monoclinic symmetry with evident orthorhombic elongation. This change yields a large uniaxial magnetocrystalline anisotropy with the easy axis in the crystallographic *c*-direction. As a result, a simple temperature scan of the magnetization, performed at a constant field, detects a very sharp increase at around 120 K because the magnetic anisotropy becomes much larger in the low temperature region. This steplike behavior can be taken as a proper fingerprint of magnetite because it disappears as soon as the stoichiometry moves away from that of pure magnetite. The position of the step can be also used to check the Fe^2+^/Fe^3+^ ratio per formula unit, as demonstrated in previous studies.^43,44^ It has been observed that the step shifts to lower temperature values as the number of Fe^2+^ decreases, either due to the Fe^2+^ vacancies in the nonstoichiometric lattice or due to the substitution of Fe^2+^ by other doping metallic ions. Any stoichiometry dispersion within the crystal brings about a fast smoothing of the transition, which can be easily quantified by the derivative of the magnetization against the temperature (the secondary *y*-axis in Figure 5).

Figure 5 presents the magnetization as a function of temperature in the range 5–300 K in zero field cooling and field cooling conditions (ZFC-FC protocol). From the Opt_1_ batch to Opt_3_ batch (that is, Figure 5a–c), both the sharpening of the Verwey transition (see the derivative of ZFC branch) and the *T*_v_ shifting to larger values (89 K in sample Opt_1_-33, 111 K in sample Opt_2_-29, and 118 K in sample Opt_3_-33) are evident. Indeed, the Fe^2+^/Fe^3+^ pairs determined by Mössbauer spectroscopy (see Table 2) present a clear lineal correlation with the *T*_v_ values subtracted from the ZFC-FC measurements (see the inset of Figure 5a). The comparison among the three batches (Opt_1,2,3_) reveals the exceptional quality of the Opt_3_ batch NPs and highlights the importance of controlling every subtlety of the chemical synthesis in order to obtain stoichiometric single nanocrystals of magnetite. As it can be seen in Figure 5c–f, the whole set of Opt_3_ batch samples present the same abrupt Verwey transition at *T*_v_ ≈ 120 K, which is, as far as we know, the first time that magnetite NPs show a *T*_v_ so similar to bulk magnetite (*T*_vbulk_ ≈ 120 K).^45^

**Figure 6:**
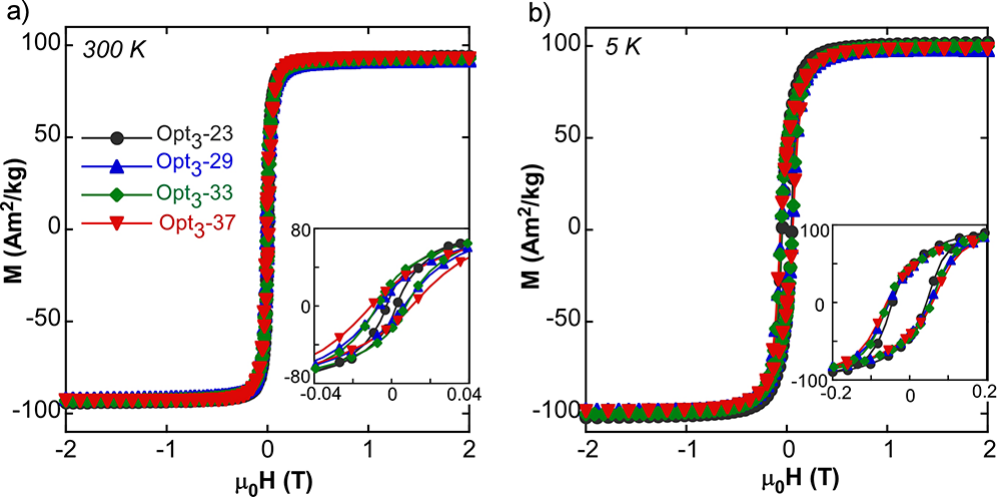
*M*(*H*) curves of Opt_3_ batch samples at (a) 300 K and (b) 5 K. The insets show the low field region.

On the other hand, Figure 6 shows the *M*(*H*) curves at RT and 5 K; the main properties of the hysteresis loops (saturation magnetization, *M*_s_, coercive field, *H*_c_, and reduced remanent magnetization, *M*_r_/*M*_s_) have been summarized in Table 3 The *M*_s_ values at RT and 5 K of the Opt_3_ batch are equal to those of pure bulk magnetite (92 and 98 A·m^2^/kg, respectively). The Opt_3_ batch not only presents almost identical *M*_s_ values to bulk magnetite but also shows a lower decrease of the magnetization from 5 K to RT (around 7–6%) compared to Opt_1_ (≈10%) and Opt_2_ (≈8%) batches (see Table 3). The almost exact match between the *M*_s_ values of the Opt_3_ batch and bulk magnetite suggests that the saturation magnetic moment of these nanoparticles, whose average dimension is above 20 nm, remains virtually undisturbed by the potential spin disorder caused by surface effects.

Hysteresis loops at 5 K (Figure 6b) show how the Opt_3_ batch reaches saturation at fields smaller than 0.5 T, meaning that the whole set of NPs are single magnetic phase objects. The shape of the hysteresis loops at 5 K fit with the Stoner–Wohlfarth model of uniaxial single domains, which predicts a reduced remanence (*M*_r_/*M*_s_) of about 0.5 (Table 3). In addition, the coercivity values at 5 K and RT are comparable to the data presented by magnetite NPs of similar sizes,^47^ and the decrease of the *H*_c_ with the lowering of the average dimension of the sample is related to the more significant thermal effects in smaller NPs. These thermal effects become way more relevant at RT affecting noticeably the hysteretic properties; as it can be seen in Table 3 and Figure 6a, both the *H*_c_ and *M*_r_/*M*_s_ of the Opt_3_ batch is progressively reduced with the decrease of the NP size.

#### 2.2.2 AC Magnetometry

**Figure 7:**
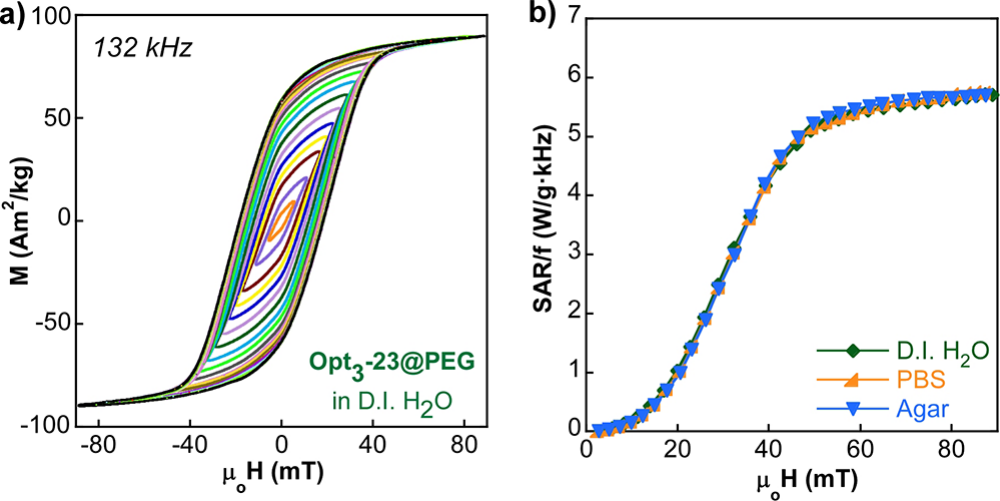
(a) AC hysteresis loops of Opt_3_-23@PEG in D.I. H_2_O at 132 kHz and (b) experimental SAR/f versus field curves of Opt_3_-23@PEG in D.I. H_2_O, in physiological media (PBS 1×), and in agar. AC hysteresis loops in PBS and agar are presented in Figure S5, Supporting Information.

An important requirement for a successful biomedical application of NP-based systems is a good colloidal stability. This requisite is specially challenging in magnetic nanoparticles (MNPs) with a permanent magnetic moment (*D* > 20 nm) because of their tendency to agglomerate due to dipolar magnetic forces,^48^ but it is even more demanding to attain stable dispersions with negligible clustering effects. Fortunately, by following a recently published coating protocol,^29^ MNP aggregation can be greatly minimized using a PMAO-PEG copolymer composed of large enough PEG tails. In fact, a good compromise is achieved using ≈25 nm magnetite NPs that do not require excessively high molecular weight (*M*_W_) PEG (5 kDa ≤ *M*_WPEG_ ≤ 10 kDa). Thus, in this section, the study of the magnetothermal efficiency of the Opt_3_ batch has been developed using the Opt_3_-23@PEG (10 kDa) sample. Figure 7a shows the AC hysteresis loops of Opt_3_-23@PEG NPs (dispersed in D.I. H_2_O) until a maximum field of 90 mT at 130 kHz. It is worthy to mention that this window of field allows for measuring the dynamical magnetization response between virtually saturated values (≈90 A·m^2^/kg). Both the shape of the loops and the characteristic curve of SAR versus AC magnetic excitation resemble others reported in the literature for magnetic single domains of magnetite NPs.^29,49^ In addition, the dependence of the rate SAR/f on the field amplitude (Figure 7b) clearly demonstrates that the magnetically driven orientation effect of magnetic easy axis that can potentially arise in colloidal samples^50^ no longer exists in this NP system. Since the hydrodynamic diameter of NP@PEG formulation is large enough (Dh > 70 nm, see Tables S2 and S3 in the Supporting Information), the heat production under an AMF is the result of the movement of the magnetic moment internally without any physical motion (i.e., Neel mechanism), which leads to almost identical AC response in D.I. H_2_O and in very viscous media (see Table S4 in the Supporting Information). Consequently, it can be stated that the high heating capacity of the Opt_3_-23@PEG sample relies entirely on its intrinsic magnetic properties. Additionally, the Opt_3_-23@PEG sample presents exceptional colloidal stability in saline media (see Table S3 and Figure S4 in the Supporting Information) in such a way that the SAR remains the same in physiological conditions, which is essential for practical hyperthermia therapies. The consistency of the heating capacity of Opt_3_-23@PEG in different environments derives from the proper surface modification that produces quasi-individually coated NPs^29^ with practically negligible dipolar interactions.

Anyways, the most crucial aspect for a successful magnetic hyperthermia treatment is to guarantee that the field and frequency conditions are clinically safe and do not lead to tissue overheating. Thus, it is essential to investigate the optimal magnetic excitation conditions that reach the maximum SAR within safety limits. Recently, Hergt et al.’s approach^51^ has been considered an acceptable threshold; according to it, the maximum field-frequency product (*H × f*) must not exceed 5 × 10^9^ A·m^–1^·s^–1^. Therefore, considering the Hergt criterion for a given field amplitude (*H*), the maximum acceptable frequency can be determined by eq 6:^47^
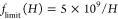
6

Therefore, the maximum achievable SAR (SAR_limit_) can be calculated to be (eq 7):
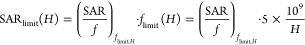
7

**Figure 8:**
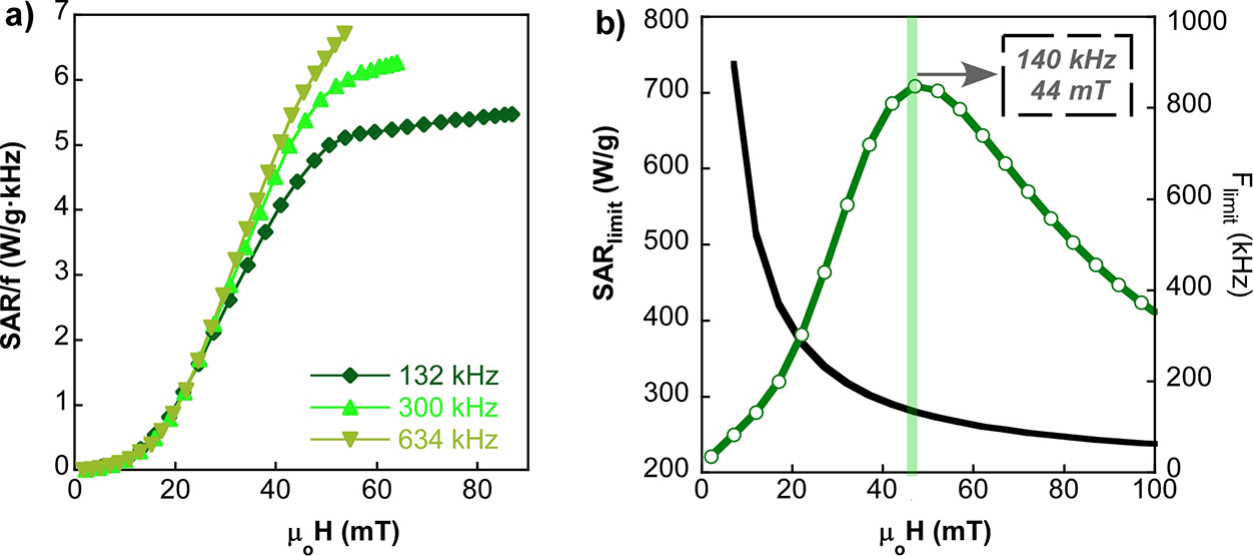
(a) Experimental SAR/f versus field curves of Opt_3_-23@PEG at different frequencies (132, 300, and 634 kHz). (b) Maximum achievable SAR and SAR_limit_ under the Hergt criterion for the sample Opt_3_-23@PEG. The black curve is the acceptable maximum frequency, *f*_limit_ (*H*) = 5 × 10^9^/*H*, for a given magnetic field intensity. The intersection of the black curve and the green bar shows the optimal conditions for obtaining the maximum SAR_limit_ value.

The mathematical reasoning of eq 7 has been applied to the sample Opt_3_-23@PEG, whose frequency-normalized SAR (SAR/f) versus field amplitude curves are not superimposed to each other as it can be seen in Figure 8a. Actually, a slightly frequency-dependent hysteresis area is expected for magnetite NPs of 23 nm,^49^ which basically means that thermal fluctuations at RT in Opt_3_-23@PEG are not negligible. Therefore, SAR/f is a function of the frequency and the field, but since the experimental data are only available for three discrete frequencies (132, 300, and 634 kHz), SAR_limit_(*H*) has been obtained by interpolation of the rest of the frequency points in the studied interval (100 kHz and 1 MHz). The current approach has allowed determining the SAR_limit_ curve for the sample Opt_3_-23@PEG, shown in Figure 8b. This SAR_limit_ curve peaks at 710 W/g for 44 mT and 140 kHz, which are the optimal excitation conditions to maximize the heating power while keeping the clinical safety. It is noteworthy that the maximum SAR_limit_ reached by Opt_3_-23@PEG is remarkably larger than other high quality ferrite NPs of similar features.^44,49^

**Figure 9:**
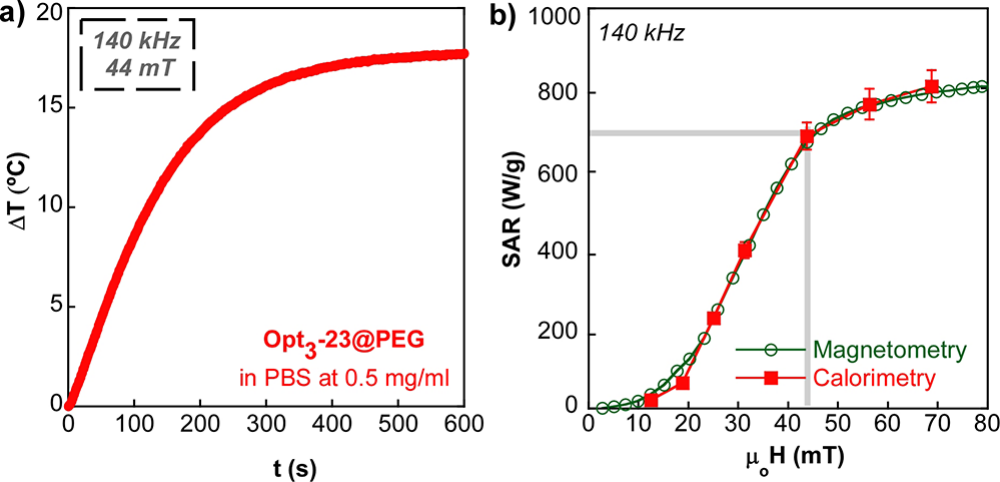
(a) Temperature increase over time at clinically safe conditions for the sample Opt_3_-23@PEG in PBS at a concentration of 0.5 mg/mL. (b) SAR vs field curves obtained by AC magnetometry and by calorimetry at 140 kHz for the sample Opt_3_-23@PEG in PBS (*c* = 0.5 mg/mL). A 5% deviation has been assumed for the estimation of the initial Δ*T*/Δ*t* slopes.

With the aim of confirming the remarkable heating power of Opt_3_-23@PEG under safe clinical conditions, the heat production of the sample in physiological media has been directly measured by calorimetry. The curve presented in Figure 9a shows the temperature increase of the sample Opt_3_-23@PEG after the onset of the safe AMF (44 mT and 140 kHz) and, as it can be observed, the *T* is boosted up to 18 °C in a 5 min interval. Moreover, the SAR values obtained from eq 8 (in the first 10 s of the experiment) at 140 kHz and different fields (12–70 mT) are completely comparable to the values calculated from AC hysteresis loops performed at the same excitation conditions (see Figure 9b).
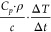
8where *C_p_* is the specific heat capacity of water, ρ is the density of water, and *c* is the magnetite NP concentration. The good match of both curves in Figure 9b does not only show the great agreement between calorimetry and AC magnetometry techniques but also reasserts the excellent heating capacity of the sample Opt_3_-23@PEG.

**Figure 10:**
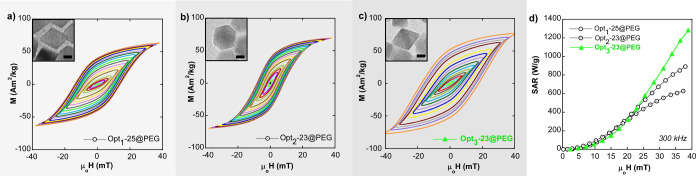
AC hysteresis loops at 300 kHz for samples (a) Opt_1_-25@PEG, (b) Opt_2_-23@PEG, and (c) Opt_3_-23@PEG. Insets: TEM micrographs, scale bars: 10 nm. (d) Experimental SAR versus field curves. The optimization stages of Opt_1_, Opt_2_, and Opt_3_ are arranged in increasing gray levels.

Finally, to better visualize the evolution of the heating capacity from the Opt_1_ batch to Opt_3_ batch, Figure 10 displays the AC hysteresis loops (at 300 kHz) of samples Opt_1_-25@PEG, Opt_2_-23@PEG, and Opt_3_-23@PEG, respectively. In this case, NPs with an average size of ≈25 nm and similar coating (PMAO-PEG) have been compared. Clearly, AC hysteresis loops of samples Opt_1_-25@PEG and Opt_3_-23@PEG, composed of octahedral particles, look rather similar to each other but quite different from that of sample Opt_2_-23@PEG, where NPs have a distinct cube-octahedral morphology. Note that coercive field values of samples Opt_1_-25@PEG and Opt_3_-23@PEG (between 15 and 20 mT) are almost twice that of Opt_2_-23@PEG, resulting in a much higher hysteresis area.

The excellent performance of octahedral magnetite particles as heat producers have been explained in previous studies as a consequence of the magnetic shape anisotropy of the octahedral morphology.^27,52^ This morphology imprints a bistable character to the anisotropy energy functional form, whose most notable effect is the increase of the squareness of the hysteresis loop (with the reduced remanence magnetization always above 0.5). The increase of SAR in the sample Opt_3_-23@PEG in comparison to the sample Opt_1_-23@PEG should be partially related to the magnetization enhancement (better stoichiometry and larger saturation magnetization, see Table 1) and, also, to the remarkable minimal agglomeration in the sample Opt_3_-23@PEG. Note at this point that the polymer coating protocol from the Opt_1_ batch to Opt_3_ batch has also been refined to minimize as much as possible the clustering effects (see hydrodynamic diameters (D_h_) in Table S2, Supporting Information). Additionally, slightly different degrees of truncation/irregularities in the octahedral-like shape of the NPs could play a perceptible role.

To put it succinctly, there are three phenomena driving the enlargement of the area under the hysteresis loops in Figure 10: (i) saturation magnetization of the NPs (*y*-axis), (ii) morphology/anisotropy of the NPs (*x*-axis), and (iii) dipolar interactions among NPs, an effect that strongly hinders the approach to saturation properties and reduces the hysteresis width (*y*–*x*-axes). Therefore, it can be roundly concluded that in the sample Opt_3_-23@PEG, the three aspects have been properly tailored to fabricate an excellent AC power nanotransducer with reproducible efficiency regardless of the viscosity and salinity of the media.

## 3 Conclusions

Fe_3_O_4_ single crystals of different sizes have been synthesized making use of a methodically improved chemical route based on the thermal decomposition of an optimized FeOl complex. A key aspect of the synthetic improvement of these magnetite NPs relies on the suitable mixture of ionic, bridging, and bidentate coordination modes in the FeOl precursor. A proper mixture of weakly and strongly coordinated ligands has been achieved by drying the precursor during 21 h, which has led to a decomposition window that enables differentiated nucleation and growth steps and gives rise to highly uniform NPs in size and shape. The refinement of other synthetic parameters (such as the amount of oleic acid and the *T* profile in the last stage of the synthesis) has also played an important role in the production of such homogeneous nano-octahedrons with unreported bulklike properties. Additionally, the average dimension of the nanoparticles has been precisely tailored (from 20 to 40 nm) by adjusting the total volume and the boiling point of the reaction mixture. The composition of the NPs has been investigated by Mössbauer spectroscopy, which has shown a perfectly stoichiometric magnetite phase. In addition, these samples have presented a saturation magnetization of 93 (2) A·m^2^/kg at RT and an extremely sharp Verwey transition at ≈120 K, which provides another explicit image of the high quality of the crystalline nanostructure. Thanks to a suitable polymeric coating around the NPs which minimizes the dipolar interactions, the heating power stays constant in different dispersion media (distilled water, agar, and physiological solution). The magnetic hyperthermia efficiency of these magnetite NPs has been studied by both AC magnetometry and calorimetry, resulting in an outstanding magnetothermal performance and a total agreement between the two techniques. The success of this NP system is the result of having tuned three crucial aspects: the saturation magnetization and the magnetic anisotropy have been maximized while the dipolar interactions have been minimized. It is worth highlighting the importance that the octahedral morphology has in the coercivity enlargement of the dynamical hysteresis loops and thus in the heating capacity of the NPs. Finally, it must be stressed that the present triumph of the chemical synthesis producing such unparalleled magnetite NPs calls into question the indefectible association between nanoparticles and defective crystal lattices.

## 4 Experimental Section

### 4.1 Materials

Iron(III) chloride hexahydrate was purchased from Acros (99%), sodium oleate from TCI America (97%), ethanol from Panreac S.A, poly(ethylene glycol)-amine (PEG-NH_2_) from Laysan Bio (*M*_W_ = 10,000), and phosphate buffered saline (PBS) from Gibco. All other solvents and reagents were purchased from Sigma-Aldrich and used as received without purification: oleic acid (90%), 1-octadecene (ODE) (90%), dibenzyl ether (DBE) (98%), hexane (99%), and poly(maleic anhydride-*alt*-1-octadecene) (PMAO) (*M*_W_ = 30,000–50,000 Da).

### 4.2 Synthesis of FeOl

For the synthesis of FeOl, 40 mmol FeCl_3_·6H_2_O and 120 mmol sodium oleate were added to a solvent mixture (140 mL of hexane, 80 mL of ethanol, and 60 mL of D.I. H_2_O) and heated to reflux (60 °C) for 1 h under N_2_ gas. After cooling to RT, the aqueous phase was removed using a separatory funnel and the organic phase containing the FeOl complex was further washed with D.I H_2_O. Finally, the organic phase with the FeOl was dried overnight at 110 °C to ensure the complete removal of hexane, EtOH, and H_2_O, resulting in a black-brownish waxy solid.

### 4.3 Synthesis of Fe_3_O_4_ NPs

In the optimized syntheses (batch Opt_3_), FeOl (5 mmol) was dissolved in a 2:1 mixture of organic solvents (ODE and DBE) together with oleic acid (10 mmol) (oleic acid:FeOl = 2:1, see Table 1). The mixture was heated in two steps under N_2_ (g) using a *T* controller (see Figure S3 in the Supporting Information): First, at 10 °C/min from RT to 200 °C and second at 3 °C/min from 200 °C to the final *T* (from 315 to 325 °C, depending on the preparation). The final temperature was adjusted by modifying the volume of the solvents and thus the boiling point of the reaction mixture. The system was kept under reflux for 60 min and then the product was cooled to RT. The entire synthesis was carried out under mechanical stirring (at 120 rpm) and by keeping the reaction flask completely sealed in order to ensure that there was no leaking. The final product was cleaned by centrifugation (20,000 rpm) using THF, EtOH, and CHCl_3_ as explained in our previous work.^27^ The stock solution was dispersed in CHCl_3_ and stored in a fridge.

### 4.4 Physical, Structural, and Magnetic Experimental Details

- FTIR spectra of FeOl complexes were collected on a FTIR-8400S Shimadzu spectrometer in a 4000–400 cm^–1^ range using KBr pellets.
- XRD patterns of the as-synthesized dried samples were obtained using a PANalytical X’Pert Pro diffractometer equipped with a copper anode (operated at 40 kV and 40 mA), diffracted beam monochromator, and PIXcel detector. Scans were collected in the 10–90° 2θ range, with a step size of 0.02° and a scan step speed of 1.25 s.
- The percentage of organic matter in the as-synthesized hydrophobic NPs was determined by thermogravimetric measurements, performed in a NETZSCH STA 449 C thermogravimetric analyzer, by heating 10 mg of sample at 10 °C/min under a dry Ar atmosphere.
- TEM micrographs were obtained using a JEOL JEM 2010 with an accelerating voltage of 200 kV and a point resolution of 0.19 nm, which provides morphology images and the corresponding crystal structures by selected-area electron diffraction.
- Mössbauer spectroscopy measurements were performed at RT in transmission geometry using a conventional constant-acceleration spectrometer with a ^57^Co-Rh source. The isomer shift values were taken with respect to an α-Fe calibration foil measured at RT. The NORMOS program develop by Brand et al. was used for fitting the spectra.
- Quasi-static magnetization measurements as a function of the magnetic field, *M*(*H*) and temperature *M*(*T*) (at 10 Oe) were carried out using a SQUID magnetometer (MPMS3, Quantum Design). These measurements were performed by diluting the as-synthesized NP stocks and depositing a drop on a semipermeable filter paper. The *M*_s_ at RT and 5 K were obtained from the dried as-synthesized samples (powder) and normalized per unit mass of inorganic matter by subtracting the weight percentage of organic matter determined by thermogravimetry.
- SAR measurements have been performed by AC magnetometry in a homemade device that generates a high magnetic field able to saturate the samples.^49^ This device is capable of working at a wide frequency range (100–950 kHz) with large field intensities: up to 90 mT at a low frequency limit and up to 31 mT at a high frequency limit. The dynamic hysteresis loops were measured at RT (25 °C) at selected frequencies of 132, 140, 300, and 634 kHz. These measurements have been carried out in PMAO-PEG-coated NPs dispersed in distilled water, in physiological media (PBS 1×), and in agar (2%). The experimental setup has been adapted to perform in situ magnetometric and calorimetric measurements. For this purpose, a sample holder includes a fiber-optic sensor immersed in the sample that allows for monitoring the temperature increase with time caused by the application of a constant radio-frequency magnetic field.

## Supporting Information Available

FTIR spectra, thermogravimetric measurements, calculation of crystallite sizes, DLS measurements, AC hysteresis loops in different media, and synthesis setup (PDF)
