# Supplementary material for: A Milestone in the Chemical Synthesis of Fe3O4 Nanoparticles: Unreported Bulklike Properties Lead to a Remarkable Magnetic Hyperthermia
Source: Chem Mater. 2021 Nov 10;33(22):8693–704. doi: 10.1021/acs.chemmater.1c02654 (PMC8619619; doi:10.1021/acs.chemmater.1c02654)
Supplement: Supplementary file 1 — cm1c02654_si_001.pdf [file cm1c02654_si_001.pdf]

SUPPORTING INFORMATION

A Milestone in the Chemical Synthesis of Fe_3O_4 Nanoparticles: Unreported Bulk-like Properties lead to a Remarkable Magnetic Hyperthermia

Idoia Castellanos-Rubio[1*], *Oihane Arriortua*[2], *Daniela Iglesias-Rojas*[2], *Ander Barón*[2], *Irati Rodrigo*[1,3], *Lourdes Marcano*[4,1], José *S. Garitaonandia*[5], *Iñaki Orue*[6], *M. Luisa Fdez-Gubieda*[1,3], *Maite Insausti*[2,3*].

[1]Dpto. Electricidad y Electrónica, Facultad de Ciencia y Tecnología, UPV/EHU, Barrio Sarriena s/n, 48940, Leioa, Spain.

[2]Dpto. Química Inorgánica, Facultad de Ciencia y Tecnología, UPV/EHU, Barrio Sarriena s/n, 48940, Leioa, Spain.

[3]BC Materials, Basque Center for Materials, Applications and Nanostructures, Barrio Sarriena s/n, 48940, Leioa, Spain.

[4]Helmholtz-Zentrum Berlin für Materialien und Energie, Albert-Einstein-Str.15, 12489 Berlin, Germany.

[5]Dpto. Física Aplicada II, Facultad de Ciencia y Tecnología, UPV/EHU, Barrio Sarriena s/n, 48940, Leioa, Spain.

[6]SGIker, Servicios Generales de Investigación, UPV/EHU, Barrio Sarriena s/n, 48940, Leioa, Spain.

Table of contents

Figure S1: FTIR spectra of FeOl annealed during 15 h, 18 h and 21 h.

Figure S2. Thermogravimetry of FeOl samples and as-synthesized NPs.

Figure S3. The synthesis setup.

Table S1. Crystallite sizes of Fe_3O_4 nanoparticles

Table S2, S3. DLS measurements: mean hydrodynamic diameter and Z potential.

Figure S4. Dh_N distribution of Opt_3-23@PEG sample over time

Table S4. Experimental viscosities of D.I. H_2O, PBS (1x) and Agar (2%).

Figure S5. AC hysteresis loops and experimental SAR(H) curves in agar and PBS.

FTIR spectra of FeOl precursors

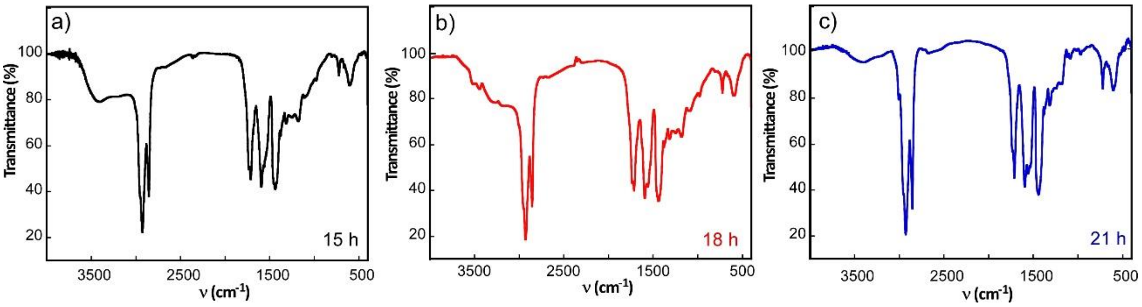

Figure S1: FTIR spectra of FeOl dried at 110 ºC during **a)** 15 h, **b)** 18 h and **c)** 21 h.

Thermogravimetric measurements

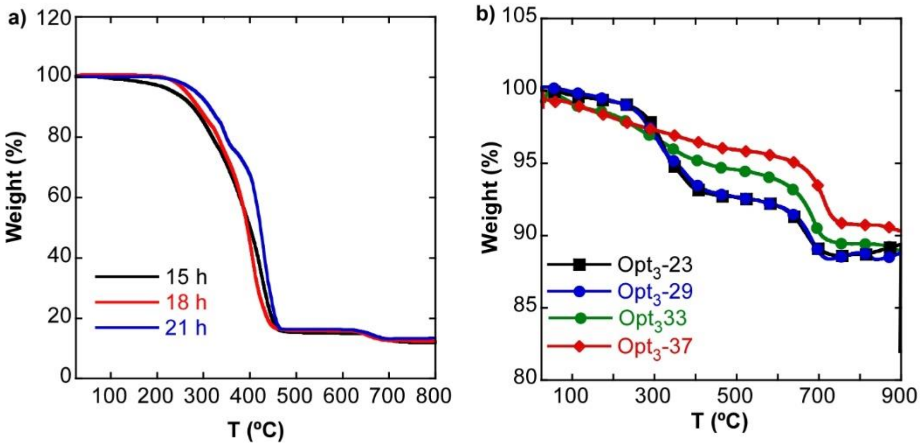

Figure S2. Thermogravimetric curves at 10 ºC/min and under Ar of **a)** FeOl samples dried during different times (15, 18 and 21 h) and of **b)** as-synthesized NPs from Opt_3-batch.

The synthesis setup

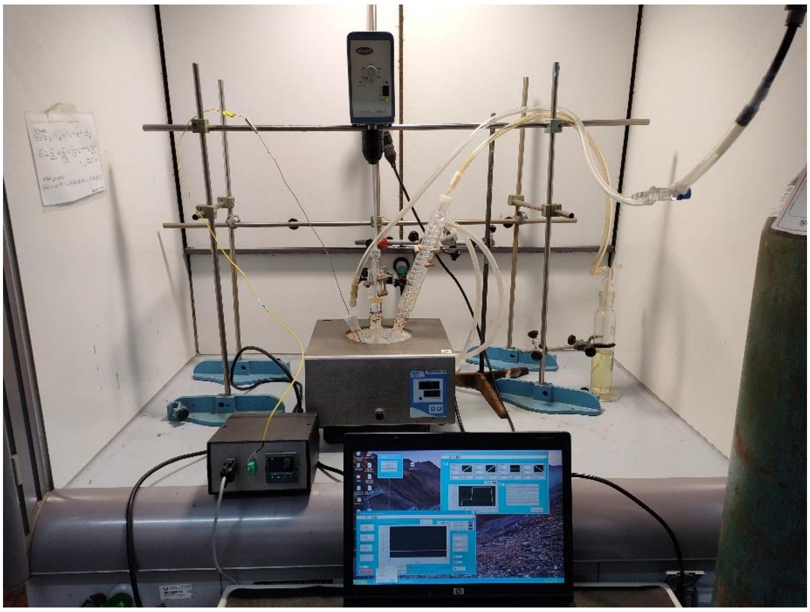

Figure S3. Set up used in the chemical synthesis of highly homogeneous and stoichiometric magnetite nanoparticles.

Crystallite size of Fe_3O_4 nanoparticles

The crystallite sizes of samples Opt_3-23, Opt_3-29, Opt_3-33 and Opt_3-37 have been calculated by the deconvolution of the (311) diffraction peak of magnetite, using the Scherrer equation (S1):

$$D = \frac{K\lambda}{B_{estruc.} \cos\theta} = \qquad \text{(S1)}$$

Where K is the shape factor (0.85-0.95), $B_{structure} = B_{observed} - B_{instrumental}$ is the full width at half maximum, λ is the X-ray wavelength (in our case = $(K\alpha_1 + K\alpha_2)/2 = 1.5418$Å), and θ is the peak position.

Table S1. Parameters obtained from the deconvolution of (311) of magnetite in samples Opt_3-23, Opt_3-29, Opt_3-33 and Opt_3-37 and the calculated crystallite size using Scherrer equation.

Sample	Diffraction peak	B obs. (°2θ)	B inst. (°2θ)	B estruc. (°2θ)	Peak pos. (°2θ)	Crystallite Size (α) (nm)*
Opt_3-23	311	0.474	0.120	0.354	35.620	24 (1)
Opt_3-29	311	0.399	0.120	0.279	35.612	30 (2)
Opt_3-33	311	0.368	0.120	0.248	35.599	34 (2)
Opt_3-37	311	0.324	0.120	0.204	35.605	40 (2)

**The deviation of the size has been obtained using K= 0.85-0.95*

Hydrodynamic-Size and Zeta-Potential

The measurement of Z Potential and hydrodinamic diameter (D_{hN}) for sample Opt_1-25, Opt_2-23 and Opt_3-23 coated with PMAO-PEG (Mw_{PEG}=10kDa) have been summarized in **Table S2**. The negative Z Potential values come from the free COOH groups in the PMAO backbone. The three samples presents very good colloidal stability with reduced clustering effects.

Table S2. Polydispersion Index (PDI), mean hydrodynamic diameter (given in Number (Dh_N) and Z potential (Pz) for sample Opt_1-25@PEG, Opt_2-23@PEG and Opt_3-23@PEG in D.I. H_2O.

SAMPLE	COATING	PDI	D_{hN} (σ)(nm) [H_2O]	Pz (σ) (mV) [H_2O]
Opt_1-25@PEG	PMAO-PEG-10K	0.41	101 (12)	-25.0 (1.0)
Opt_2-23@PEG	PMAO-PEG-10K	0.29	78 (5)	-8.0 (0.4)
Opt_3-23@PEG	PMAO-PEG-10K	0.25	76 (5)	-16.0 (0.9)

Sample Opt_3-23@PEG presents the best stability and a remarkably minimal agglomeration (D_{hN} < 100 nm) in both distillate water and physiological conditions (PBS). Sample Opt_3-23@PEG dispersed in H_2O and in PBS (1x) has been stocked in the fridge for over a year and its colloidal properties have been studied.

The DLS analysis of Opt3-23@PEG in 04-06-2020 and in 14-06-2021 are quite equivalent (see **Table S3**), which proves the excellent long-term stability of these nano-platforms both in D.I. H_2O and in physiological conditions (PBS). In order to better illustrate the small changes of the colloidal properties of Opt3-23@PEG sample over time, the Dh_N distributions obtained in 2020 and in 2021 have been plotted together and are displayed in **Figure S4**.

Table S3. Mean hydrodynamic diameter given in Intensity (Dh_I), Volume (Dh_V) and Number (Dh_N)) in D.I. H_2O and in PBS (1x) and Z potential (Pz) in D.I. H_2O for sample Opt3-23@PEG. The set of measurements have been repeated one year later to analyze the colloidal stability of the sample.

SAMPLE	Disersion media	DLS (nm) D_h (σ)			Pz (σ) (mV) [H_2O]	Date
		Intensiy	Volume	Number		
Opt_3-23@PEG	H_2O	202 (10)	111 (6)	76 (5)	-16 (1)	04-06-2020
	PBS	199 (8)	122 (4)	88 (4)		
Opt_3-23@PEG	H_2O	110 (6)	100 (3)	89 (4)	-15 (1)	14-06-2021
	PBS	124 (3)	112 (2)	98 (2)		

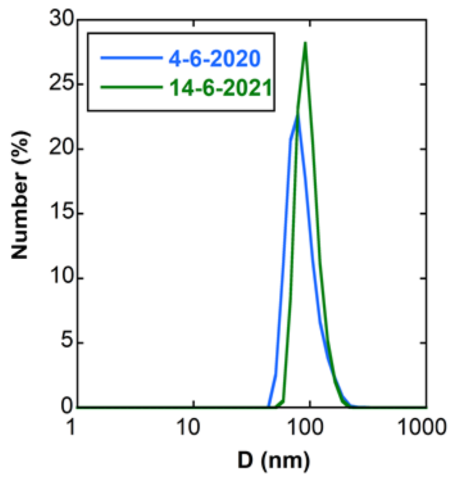

Figure S4. Dh_N distributions of Opt_3-23@PEG sample dispersed in PBS (1x) in 04-06-2020 and in 14-06-2021.

Viscosity (µ) of the dispersion media.

Opt_3-23@PEG sample has been dispersed in three different media:

1) Distillate water (D.I. H_2O, pH=6)
2) Phosphate-buffered saline (PBS, 1x, pH=7.4)
3) Agar aqueous solution prepared at 2% (w/v).

In order to disperse the NPs in agar, the agar solution is heated to boiling and then the NPs are added. The mixture is stirred until a homogeneous NP-agar dispersion is achieved. Finally, the mixture is cool down to room temperature (RT), where the agar is in solid state presenting a very high viscosity ($\mu \rightarrow \infty$). The gelling temperature of the agar (2%) is ≈40 ºC. The viscosity values of the media have been obtained making use of an AMETEK Brookfield DVST Viscometer and have been summarized in **Table S4** at RT and 50ºC.

Since AC magnetometry is carried out at RT, the NPs are immobilized within the solid agar matrix during the measurement. Consequently, it can be concluded that in this media physical orientation effects are impeded.

Table S4. Experimental viscosities (cP) of the three dispersion media: D.I. H_2O, PBS (1x) and Agar (2%).

Media	T= 22°C	T= 50°C
**D.I. H_2O**	1.11	0.95
**PBS (1x)**	1.26	0.85
**Agar 2%**	$\rightarrow \infty$	31.75

**gelling T of agar (2%)≈ 40 ºC*

AC loops of sample Opt$_3$-23@PEG in physiological solution and agar

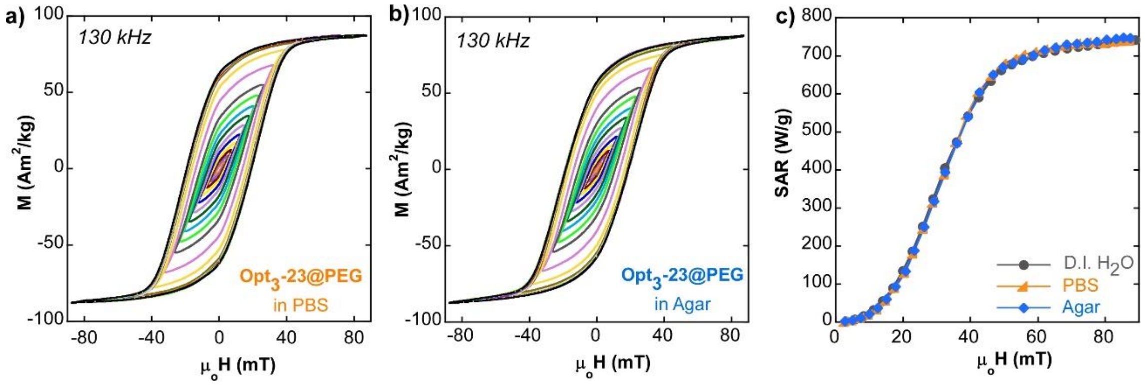

Figure S5. AC hysteresis loops of Opt$_3$-23@PEG in **a)** physiological solutions (PBS x1) and **b)** agar at 132 kHz. **c)** The corresponding experimental SAR vs field curves.
